# Varied Presentations of Pediatric Patients With Positive Cannabinoid Tests

**DOI:** 10.7759/cureus.23493

**Published:** 2022-03-25

**Authors:** Taylor Harvey, Ramon Gomez, Brian Wolk, Ali Ozcan

**Affiliations:** 1 Emergency Medicine, Loma Linda University Medical Center, Loma Linda, USA; 2 Emergency Department, University of California Riverside, Riverside, USA; 3 Pediatric Emergency Medicine, Rady Children's Hospital, San Diego, USA

**Keywords:** intoxication, pediatric, cannabinoid, marijuana, cannabis

## Abstract

Background: Cannabis (marijuana) is one of the most abused drugs worldwide. The increasing availability of cannabis has been associated with increased emergency department (ED) visits. There is a wide range of presentations documented in the recent literature, and the full scope of symptoms in young children is not fully known.

Objective: The primary objectives were to gather information regarding the characteristics in the presentation of the children with positive cannabinoid urine drug screen (UDS) results in the ED and to determine if there are certain common presentations with cannabinoid ingestion or inhalation.

Design/methods: This study was a descriptive retrospective chart review from March 2013 to June 2020 of pediatric patients <18 years old with positive UDS for cannabinoids. Data collected included age, gender, chief complaint, history, review of systems, vital signs, physical exam findings, laboratory studies, imaging findings, and disposition. Four hundred and twenty-two charts were included in the study. Analysis was done using Stata 13 (College Station, TX).

Results: The data showed that there was a significant increase in the number of pediatric patients with a positive UDS after cannabis legalization. Using cases from November 2013 to November 2019 showed 71% of cases presented after legalization on November 8, 2016 (Z=7.72, p<.01). The majority of cases were patients between the ages of 15 and 17 (78%). 43% (n=182) of patients presented with chief complaints of suicidal ideation. The other most common chief complaints were vomiting (8%, n=33), nausea (5%, n=22), trauma (5%, n=21), and altered mental status (AMS) (5%, n=20). The most common vital sign abnormalities included tachycardia (27%, n=115) and hypertension (18%, n=74). Forty-two percent of patients had tests ordered during their visit with 7% undergoing head computerized tomography. On the UDS, 28% of patients were positive for at least one other drug with amphetamine being the most common (13%, n=55).

Conclusion: Our data showed a significant increase in the number of cases since the legalization of cannabis in 2016, supporting the need for ED physicians to become more familiar with cannabis intoxication and its complications. The presentations of these patients can vary greatly. Common presentations include suicidal ideation, nausea/vomiting, AMS, and trauma with vital sign abnormalities including tachycardia and hypertension. Physicians should continue to consider cannabis use when evaluating these pediatric complaints. It may decrease the number of tests ordered in this patient population.

## Introduction

Cannabis (marijuana) is one of the most abused drugs worldwide. The past decade has seen an increase in the legalization of cannabis for both medicinal and recreational use. As of June 2021, 36 states allow the use of cannabis for medicinal purposes, and that number is expected to increase with additional states considering legalization in the near future [1]. The increasing availability of cannabis has been associated with increased emergency department (ED) visits in pediatric cannabis exposures for both accidental and intentional ingestions [2]. Particularly among young adults, the rate of cannabis use is increasing. It is now the most common illicit drug used by adolescents in the United States [3]. This may be partially due to adolescents perceiving cannabis as “low risk,” with only 24% of children ages 12-17 years believing cannabis use is risky [4].

Given the increasing use of cannabis, it is more important than ever for pediatricians and pediatric emergency physicians to be familiar with cannabis intoxication and its presentation in pediatrics. There is a wide range of presentations documented in the recent literature, and the full scope of symptoms in young children is not fully known. Additional research on pediatric exposures to cannabis is needed to fully describe the key signs, symptoms, and outcomes of pediatric cannabis cases.

Our study aims to gather information regarding the characteristics in the presentation of the children with positive cannabinoid urine drug screen (UDS) results in the ED and to determine the common chief complaints, presentations, and physical exam findings associated with cannabinoid ingestion or inhalation. Therefore, our study may increase awareness in the pediatric and pediatric emergency world.

## Materials and methods

Protection of human subjects

The Institutional Review Boards (IRB) for Loma Linda University Medical Center approved this study (IRB # 5200207). Informed consent was waived. 

Setting

This study was a descriptive retrospective chart review at Loma Linda University Medical Center, a tertiary hospital that has 18 pediatric emergency beds and sees around 45,000 pediatric patients a year. 

Data collection and statistical analysis

**Figure 1:**
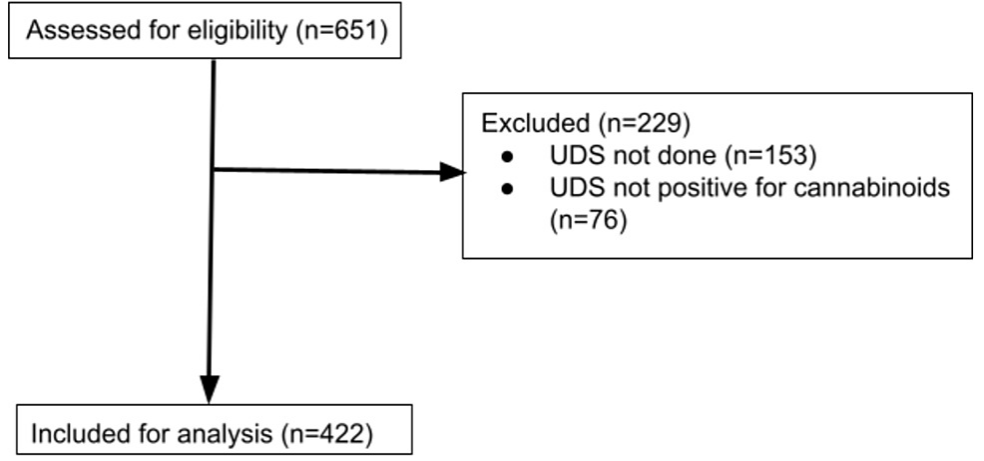
Consort Flow Diagram

To identify relevant cases from patient charts, the study team utilized an analytics function of EPIC electronic medical records. The charts collected ranged from March 2013 to June 2020. Inclusion criteria consisted of pediatric patients <18 years old evaluated in the ED with positive UDS for cannabinoid identified using ICD-10 codes. Data collected included age, gender, chief complaint, history, review of systems, vital signs, physical exam findings, laboratory studies, imaging findings, and disposition. The patient’s highest and lowest recorded vital signs (heart rate, systolic blood pressure, respiratory rate, and oxygen saturation) were abstracted from the ED documentation for each case, and vital sign abnormalities were identified using age-appropriate normal vital sign ranges defined by Tintinalli’s Emergency Medicine. Cases identified by this algorithm were manually reviewed by the principal investigator, TH (PI) to determine whether they met inclusion or exclusion criteria. Exclusion criteria included no UDS done or UDS negative for cannabinoids. A total of 651 charts was initially reviewed. Of those, 151 were excluded for not having a UDS collected during their ED visit, and 76 were excluded for having a negative UDS for cannabinoids. Also, 422 charts were included in the study (Figure 1). Analysis was done using Stata 13 (College Station, TX).

## Results

**Figure 2:**
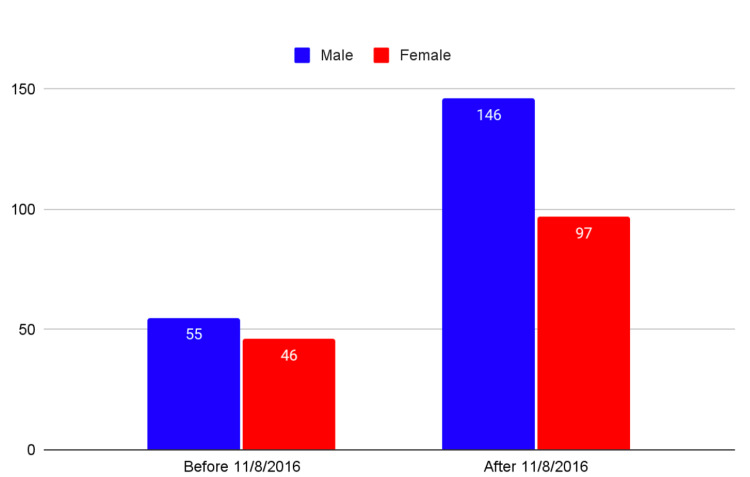
Pre/post cannabis legalization positive cannabinoid in pediatric patients

The data showed that there was a significant increase in the number of pediatric patients with a positive UDS after cannabis legalization in California on November 8, 2016. Analyzing cases from November 8, 2013 to November 8, 2019 showed 71% of cases presented after legalization on November 8, 2016 (Z=7.72, p<.01) (Figure 2).

The majority of cases were patients between the ages of 15 and 17 (78%) with less than 1.5% cases occurring in patients <10 years old. Though not statistically significant, there was a higher incidence of cases in winter (28%, n=99) and spring (27%, n=95) compared to summer (22%, n=76) and fall (23%, n=81). 43% (n=182) of patients presented with chief complaints of suicidal ideation.

The other most common chief complaints were vomiting (8%, n=33), nausea (5%, n=22), altered mental status (AMS) (5%, n=20) and trauma (5%, n=21). The most common vital sign abnormalities included tachycardia (27%, n=115), hypertension (18%, n=74), and tachypnea (16%, n=69). 42% of patients had tests ordered during their visit with 7% (n=31) undergoing head computerized tomography.

On the UDS, 28% of patients were positive for at least one other drug with amphetamine being the most common (13%, n=55). Forty-two percent (n=176) of patients were able to be discharged home and 51% (n=215) were admitted to an inpatient psychiatric facility. The most common diagnosis on completion of the visit was suicidal ideation (44%, n=187) and drug use (26%, n=109).

Patients <10 years Old with positive UDS for cannabinoid

We had a total of eight cases with patients 10 years of age or younger with a positive UDS for cannabis. In these eight cases, the ages ranged from 13 months to 10 years old. Five of the patients had a chief complaint of AMS. The other chief complaints were non-accidental trauma (NAT) evaluation, drug overdose, and abdominal pain, emesis in one patient each. 

Of these cases, seven patients had vital sign abnormalities. Six patients experienced tachycardia, two experienced tachypnea, one was febrile, and one had hypotension. 

All eight of these patients had extensive lab testing and imaging. All of them had at minimum a complete blood count (CBC) and comprehensive metabolic panel (CMP) order. Four of them had additional blood work including alcohol, acetaminophen, and salicylate levels orders. Five of them underwent computerized tomography of the head (CTH). 

In terms of disposition, four of these patients were discharged home from the ED, two were transferred to an outside hospital, and two were admitted to the children’s hospital. 

Seven out of eight cases were due to accidental ingestions. Three cases involved patients eating edible candies/baked goods belonging to a family member. One case involved accidental inhalation from a vape pen. One case involved ingestion of cannabis oil. In the remaining three cases, the source of ingestion was unknown (Table 1). For example, the 13-month-old patient presented with AMS and she was waking up easily with tactile stimulation. The only abnormal vital sign was tachycardia. Due to the AMS, the patient had a workup done for possible ingestion, head trauma, electrolyte abnormalities including hypoglycemia. All laboratory and imaging studies were normal except for positive cannabinoids in UDS. During the ED stay, the patient was more awake, after social work consultation, the patient was discharged home.

**Table 1:** Patients < 10 years old with positive cannabinoid NAT, non-accidental trauma; CBC, complete blood count; CMP, comprehensive metabolic panel; AMS, altered mental status

Age	Chief Complaint	Vital Sign Abnormalities	Tests ordered	Disposition	Type of Ingestion
13m	AMS	Tachycardia	CBC,CMP, Alcohol level,salicylate level, acetaminophen level, glucose, CT head	Discharged home	Unknown ingestion
2yo	Drug Overdose	Tachycardia, febrile	CBC,CMP, Alcohol level,salicylate level, acetaminophen level, glucose, CT head	Admitted	Accidental ingestion - vape pen
3yo	NAT evaluation	Tachycardia, tachypnea	CBC,CMP, CT head	Discharged home	Marijuana oil ingestion
3yo	AMS	Tachycardia	CBC,CMP	Discharged home	Accidental ingestion - edibles
5yo	Fatigue, abdominal pain, emesis	Tachycardia, tachypnea	CBC,CMP, CT head	Discharged home	Unknown ingestion
7yo	Seizures, AMS	None	CBC,CMP, glucose	Admitted	Unknown ingestion
8yo	AMS	Tachycardia, hypertension	CBC,CMP, Alcohol level,salicylate level, acetaminophen level, glucose, CT head	Transfer to another hospital	Accidental ingestion- edibles
10yo	AMS	Hypotension	CBC,CMP, Alcohol level,salicylate level, acetaminophen level, glucose, CT head	Transfer to another hospital	Accidental ingestion- edibles

## Discussion

Our data showed a significant increase in the number of cases since the legalization of cannabis in 2016, supporting the need for ED physicians to become more familiar with cannabis intoxication and its complications. Increased pediatric exposure to cannabis has been concurrent with the rise in adult cannabis use, which has more than doubled over the past decade [2]. The use of cannabis among parents with children in the home has increased [5], which may also contribute to the risk of accidental ingestion of cannabis products by children due to increased accessibility. This was true of our patients <10 years old, with four out of eight cases being due to accidental ingestion of a parents’ cannabis product. The presentations of these patients can vary greatly. Common chief complaints include suicidal ideation, nausea/vomiting, AMS, and trauma with vital sign abnormalities including tachycardia, tachypnea, and hypertension.

Nausea and vomiting in particular are consistent with both acute toxicity and the known phenomenon of cannabinoid hyperemesis syndrome (CHS). The common features of the CHS patient presentation include severe cyclic nausea/vomiting, relief with external thermoregulation, epigastric/periumbilical pain, and minimum frequency of weekly use of cannabis [6]. Early recognition of this syndrome may help avoid unnecessary laboratory and imaging to assess for another cause.

Cannabis is known to have psychoactive properties and has been associated with increased anxiety, paranoia, and cognitive dysfunction [7]. Among patients that presented to our ED with positive UDS for cannabis, a large proportion of these patients presented with a chief complaint of suicidal ideation and complaints of anxiety and depression. In an adolescent cohort 12-15 years of age who presented with suicidal ideation, cannabis use was associated with an increased likelihood of a suicidal attempt [8]. A study from Colorado evaluating mental health visits in the ED showed that the prevalence of mental health conditions with cannabis-associated diagnostic codes is higher than those without cannabis [9]. From a pediatric ED perspective, cannabis use should be considered for patients who present with psychiatric symptoms including anxiety or psychosis. In addition, patients who test positive for cannabis use should be screened for depression and suicidal ideation.

Another common chief complaint from our cohort was trauma. Cannabis use can lead to increased physical injuries as ingestion can decrease cognitive, perceptive, and psychomotor function. Most injuries are unintentional accidents such as falls or motor vehicle collisions. Cannabis intoxication affects judgment in the adolescent population that can lead to an increase in overall risky behaviors [10]. In 2017, the Centers for Disease Control and Prevention Youth Risk Behavior Survey found that 13% of youth reported driving after using cannabis [11]. Although driving under the influence of alcohol is known by many adolescents to be a dangerous behavior, there may not be as much awareness that cannabis intoxication can cause adverse effects on driving abilities as well.

Physicians should continue to consider cannabis use when evaluating these pediatric complaints. Awareness of the spectrum of symptoms of cannabis ingestion in a pediatric patient can help guide an evaluation and may prevent a costly and invasive workup in a child who presents with these symptoms.

Limitations of this study include the fact that the mechanism of cannabis use is unknown (inhaled vs. edible). Presentations could differ based on if the patient is smoking cannabis vs. eating edibles containing tetrahydrocannabinol (THC). Edible products contain variable amounts of THC with multiple servings sometimes combined in one product (one cookie, brownie, etc.). It would be helpful to know the mechanism of intoxication to differentiate if certain symptoms are more associated with inhalation vs edibles. In addition, the dosing/strength of each incident is unknown. If the patients were using edibles, some products contain as much as 500mg of THC per package [12]. Therefore, a single serving taken by a child could result in more severe symptoms and toxicity.

Another difficulty with utilizing the UDS for cannabis cases is the uncertainty of when the patient used cannabis last. UDS can detect cannabis in the urine for up to two weeks after the patient used cannabis, sometimes longer if the patient is a chronic user [13]. In accidental ingestions in younger children, they are more likely to present soon after the ingestion with the onset of symptoms. Young children with cannabinoid toxicity usually have been exposed to high concentration products, such as edibles, resins, or vaping fluid [14]. Adverse effects of using cannabis include distorted perception, poor concentration, psychosis, excessive vomiting, and addiction [15]. In the adolescent pediatric patient who is using cannabis recreationally, there may be hesitation to admit to intentional cannabis usage due to fear of their parents or medical staff. This can make determining an accurate timeline of cannabis use in relation to presentation to the ED more difficult.

## Conclusions

In our study, it was shown that there was a significant increase in the number of cases with positive cannabinoid tests. With proper education by primary care providers, the numbers could be decreased in the future. Advising to keep any cannabinoid product out of reach of pediatric patients, in a locked cabinet or a safe, would decrease accidental ingestions. Explaining the negative effects of cannabinoids on teenagers might help to decrease overdoses. Cannabis intoxication symptoms and presentations may vary greatly with the age groups and may include suicidal ideation, nausea/vomiting, AMS, and trauma with vital sign abnormalities including tachycardia and hypertension. Therefore, physicians should become more familiar with cannabis intoxication and its complications. Physicians should continue to consider cannabis use when evaluating these pediatric complaints. It may decrease the number of tests ordered in this patient population.
